# Differential contributions of subregions of medial temporal lobe to memory system in amnestic mild cognitive impairment: insights from fMRI study

**DOI:** 10.1038/srep26148

**Published:** 2016-05-17

**Authors:** Jiu Chen, Xujun Duan, Hao Shu, Zan Wang, Zhiliang Long, Duan Liu, Wenxiang Liao, Yongmei Shi, Huafu Chen, Zhijun Zhang

**Affiliations:** 1 Department of Neurology, Affiliated ZhongDa Hospital, School of Medicine, Southeast University, Nanjing, Jiangsu 210009, PR China; 2 Key Laboratory for NeuroInformation of Ministry of Education, School of Life Science and Technology, University of Electronic Science and Technology of China, Chengdu 610054, PR China; 3 Department of Psychology, Xinxiang Medical University, Xinxiang, Henan 453003, China

## Abstract

Altered function of the medial temporal lobe (MTL) is a valuable indicator of conversion from amnestic mild cognitive impairment (aMCI) to Alzheimer’s disease. This study is to delineate the functional circuitry of multiple subdivisions of parahippocampal gyrus and hippocampus (HIP) and to examine how this knowledge contributes to a more principled understanding of the contributions of its subregions to memory in aMCI. The functional connectivity (FC) analysis was performed in 85 aMCI and 129 healthy controls. The aMCI demonstrated the distinct disruptive patterns of the MTL subregional connectivity with the whole-brain. The right entorhinal cortex (ERC) and perirhinal cortex (PRC) showed increased connectivity with the left inferior and middle occipital gyrus, respectively, which potentially indicated a compensatory mechanism. Furthermore, the right altered MTL subregional FC was associated with episodic memory performance in aMCI. These results provide novel insights into the heterogeneous nature of its large-scale connectivity in MTL subregions in memory system underlying the memory deficits in aMCI. It further suggests that altered FC of MTL subregions is associated with the impairment of the differential encoding stages of memories and the functional changes in the specific right HIP-ERC-PRC-temporal circuitry may contribute to the impairment of episodic memory in aMCI.

Amnestic mild cognitive impairment (aMCI), which is characterized by the impairment of episodic memory, is a high rate of conversion to Alzheimer’s disease (AD)^1^,^2^. In humans, monkeys, and rats, the memory system of medial temporal lobe (MTL) is essential to declarative memory processes underlying remembrance for new events and facts^3^. The MTL is not only one of the earliest brain regions to present with pathology that leads to memory impairment, a hallmark of AD, but its subregions have a selective topography of pathological involvement during early disease^4^. Therefore, it is critical to promote our understanding of abnormalities of the function of the MTL memory system early in the course of AD.

Growing evidence suggests a more differentiated picture, one of functional diversity in the MTL subregions^5^,^6^. The monkeys and rodents studies delineate that the perirhinal cortex (PRC) receives projections largely from inferior temporal cortex areas, and projects them to the lateral entorhinal cortex (ERC)^7^. Numerous animal lesions and human fMRI studies have indicated that the PRC is related to visual object recognition memory^8^,^9^. The parahippocampal cortex (PHC) receives projections from the lateral and posterior-medial parietal cortex, and projects them to the medial ERC^7^. Findings from primate and human functional imaging studies have demonstrated that PHC damage is associated with impaired recognition of spatial and navigational information^10^,^11^. Inputs into the ERC converge from the PRC and the PHC within the MTL^12^. Due to the afferent and intrinsic pattern of ERC connectivity, the anterolateral and posteromedial ERC are related to a relative segregation of object and spatial information processing, respectively^13^. Impairment of the entire human ERC is associated with episodic memory dysfunction^14^. Both the lateral and medial ERC projections converge into the HIP at the top of the MTL cortical processing hierarchy^7^,^13^. Evidence from animal and human studies indicates that the HIP in combination with the ERC, binds information across temporal and spatial intervals, eventually forming multi-componential semantic and episodic memories^5^,^12^. Moreover, several human studies have also shown different anatomical and functional connectivity (FC) of the MTL subregions^15^,^16^,^17^,^18^. Taken together, these observations suggest that while the entire network of highly interconnected subregions is involved in declarative memory formation, each subregion may be specialized to process a unique aspect of the event or concept^19^.

Recently, several neuroimaging evidences indicate that aMCI can be characterized by abnormalities in resting-state FC of MTL subregions^20^,^21^,^22^,^23^. However, these above-mentioned studies do not explore the full-scale information of whole brain, or manually draw regions of interest (ROIs), or only provide results in coarsely divided subregions of the HIP and a limited view of MTL circuitry. Furthermore, these studies are performed in a relatively small cohort, which may have influenced statistical power or sensitivity resulting in the identification of additional areas of abnormality to some degree. Nonetheless, converging findings suggest that the deficits of MCI-spectrum subjects seem to be associated with overall MTL subregions, which suggests that there can be the distinct disrupted large-scale organization and FC profiles of MTL subregions in aMCI. The distinct functions of these areas are thought to arise from differences in local circuit properties and their interactions with distributed brain areas^24^. However, very little is known about the distinct disrupted functional organization and FC of the MTL subregional networks at the whole-brain level in aMCI. Therefore, measures of MTL function, such as FC, obtained within specific subregions of MTL may potentially be more sensitive to early disease stages.

The objective of the present study was therefore to investigate the large-scale functional organization and functional circuitry of MTL subregional networks in aMCI. We hypothesized that aMCI would present differentially abnormal connectivity patterns in the MTL subregional networks compared to healthy controls (HC). And we further predicted that the altered FC in a specific pathway along the MTL cortical processing hierarchy system would link to the impairment of episodic memory in aMCI. To answer this issue, the present study evaluated the connectivity patterns based on two parallel arrays of seeds along the longitudinal axis of the parahippocampal gyrus (PHG) and the HIP in each hemisphere (Fig. 1A,B for detailed data analysis pipeline).

## Results

### Demographic and neuropsychological characteristics

Demographic and neuropsychological characteristics are shown in Table 1. No significant differences in age, gender, or years of education were found between aMCI and HC (all *p* > 0.05). Compared with HC, aMCI showed significantly lower MMSE and MDRS scores, and significant deficits in performance in multiple-domains of cognitive functions, including episodic memory, information processing speed, executive function, and visuospatial cognition (all *p* < 0.05). Detailed raw scores and the corresponding Z scores from individual neuropsychological tests are provided in Table S1.

### Distinct functional connectivity networks of MTL subregions

FC patterns of each MTL subregion and correlation matrix within MTL subregions are shown in Figs S1–S3. Both in aMCI and HC, spontaneous activity in the MTL subregions strongly correlated with activities in a widely-distributed set of cortical and subcortical regions, as well as cerebellum (see details in *SI Results*). Furthermore, schematic polar plots illustrated that there were different FC patterns among four PHG and three HIP seeds, which showed similar patterns between aMCI and HC by visual inspection (see Fig. 2).

### Heterogeneous altered FC along anterior to posterior axis of PHG and HIP with target networks

As shown in Fig. 3, several regions showed abnormal connectivity in the MTL subregional networks in aMCI compared to HC. The detailed brain regions on distinct disrupted FC along the anterior to posterior axis of PHG and HIP are provided in Tables 2 and 3.

In the MTL subregional networks, aMCI demonstrated the distinct disrupted FC with the cortical and subcortical regions. aMCI showed a trend toward decreased FC with fusiform gyrus, precuneus, cuneus, angular gyrus, thalamus, temporal lobe, and cerebellum. Interestingly, in the right ERC and PRC networks, aMCI showed increased FC with the left occipital gyrus (see Fig. 3, and Tables 2 and 3).

These disrupted FC maps in aMCI compared to HC were superimposed to illustrate their overlap in PHG and HIP seeds. No overlapping regions of altered FC were found in different MTL subregional networks (see Fig. 3).

### Behavioral significance of the disrupted functional connectivity of MTL subregional networks

The multiple linear regression analysis demonstrated that the mean altered FC strength between right ERC and HIP, between right PRC and HIP, and between right PRC and ITG closely correlated with episodic memory composite Z score in aMCI (see Fig. 4A,B). Additionally, no correlations were evident with respect to other cognitive functions in aMCI. However, no significant correlations were found between the FC values in the same regions with cognitive performance in HC.

### Independent associations between the aging process and increased FC in aMCI

The curve estimation demonstrated that the relationships between the mean increased FC strength (mean FC between right ERC and left LOG, and right PRC and left MOG) and age were quadratic, namely inverse U-shaped curve in aMCI (see Fig. 4A,C). Furthermore, the peak of the curves was estimated to locate at around 70 years old. However, the age-related resting patterns were not found in HC.

## Discussion

The present study identified a distinct altered FC pattern associated with cognitive impairment and a selective topography of functional changes in the MTL subregional networks, indicated that the disruption of the right HIP–ERC–PRC–temporal pathway was responsible for the impairment of episodic memory, and provided new insights to understand the pathophysiology underlying the memory deficits in aMCI.

### Different FC patterns of the MTL subregions

The present study indicated that the MTL subregions showed different patterns of connectivity with cortical and subcortical areas, which corroborated and extended findings of functional heterogeneity along the longitudinal axis of the HIP and PHG in both animals^19^,^25^ and humans^18^,^26^. And these findings underlined key aspects of functional heterogeneity within the human MTL^17^,^18^,^27^,^28^,^29^, which suggests that each MTL subregion performs different specific functions linked to different forms of memory-guided behavior, respectively^5^,^6^,^19^,^25^.

### Altered functional connectivity of MTL subregions in aMCI compared to HC

Our results showed that there were differentially abnormal connectivity patterns along the anterior to posterior axis of PHG and HIP and the asymmetry of damaged degree between left and right hemispheres in aMCI compared to HC (see Figs 2A,B and 3C, Fig. S1). Our findings suggest that MTL subregions may have a selective topography of pathological involvement in aMCI^23^,^30^,^31^,^32^.

Based on our findings, as well as these findings from structural connectivity, as indicated by tract-tracing in animals^7^,^13^ and diffusion tensor imaging (DTI) in humans^16^,^33^, FC^17^,^18^, and task-sate fMRI studies^5^,^7^, we propose the following speculative model (see Fig. 5) to explain how altered connectivity patterns of MTL subregions contribute to a more principled understanding of the contributions of its subregions to memory in aMCI. As shown in Fig. 5, the schematic drawing represents the information processing that the MTL memory system processes perception information into memory^24^,^34^. This system clearly shows that two neuropathways are needed to complete the formation of memory: occipito-temporal visual object processing pathway (the “what” stream)^7^,^25^ and parieto-temporal visuospatial pathway (the “where” stream)^35^,^36^. In addition, numerous studies on the functional organization of the MTL also focus on relative differences between dorsal and ventral stream visual inputs from two distinct neocortical networks^5^,^7^,^17^,^18^,^37^,^38^,^39^,^40^.

As shown in Fig. 4, the present study indicated that the PRC network in aMCI exhibited mainly decreased connectivity with the right inferior temporal gyrus. Evidence from primate and human neuroimaging studies has indicated that these connectivities exactly locate in the occipito-temporal visual object processing pathway of two parallel pathways within the MTL memory system^7^,^17^,^18^. The inferior temporal lobe has been considered as a connectional “hub” linking sensory information about the meaning of words, objects, facts and the hippocampus via PRC^17^. It has been established that the PRC represents the apex of this pathway, which is responsible for complex integration of visual object features^19^. In line with this connectivity pattern, numerous animal lesions and human fMRI studies have demonstrated that the PRC plays a key role in multimodal object memory^25^, visual object recognition memory^41^, and semantic object memories^7^,^19^. Although the PRC network in aMCI exhibited increased connectivity with the left middle occipital gyrus, it suggests a maladaptive and/or pathogenic mechanism, which may reflect an unsuccessful attempt to recruit preserved neuronal areas to compensate for pathology, as well as an imbalance between inhibition and excitation in impaired networks owe to impending pathological processes^42^,^43^. Taken together, these findings further suggest that the aMCI may present an impairment on the formation of visual and multimodal memories of meaningful objects, that is, semantic object memories^7^,^19^.

Our results showed that the pPHC network in aMCI exhibited decreased connectivity with parietal lobe (cuneus, precuneus, and angular gyrus) and middle temporal gyrus, which locate in the parieto-temporal visuospatial pathway^35^,^36^. It has been established that PHC connected with a posterior cortical network^17^,^18^, and the PHC, retrosplenial, and medial and ventral parietal cortices correspond to a cortical network that is interconnected via the cingulum bundle^33^. It has been reported that the PHG is thought to be the primary DMN node in the MTL that mediates the FC between the DMN and MTL subregions involved in the formation of memory^44^. We also found that decreased FC to the pPHC network was involved in the DMN regions in aMCI patients. Numerous studies have supported that Aβ deposition first occurs within the DMN in aMCI patients^45^,^46^ and is associated with impaired resting state connectivity^47^,^48^. The present study provides further evidence for the disconnection of the DMN in aMCI^49^,^50^, which is especially associated with the pPHC network. And notably, the aPHC network in aMCI only showed reduced connectivity with middle temporal gyrus, which suggests that aPHC and pPHC may be two distinct subregions within the MTL^17^. Indeed, previous fMRI studies have also observed that posterior parts of the bilateral PHC more process both perceptual and mnemonic features of scenes, that is, the visuospatial arrangement of landmarks, which enable orientation and navigation in the environment^19^,^51^. Therefore, these findings may be partially responsible for the unable orientation and navigation in the environment, a key clinical symptom in late stages of disease progression of aMCI.

The present study showed that the ERC network mainly exhibited decreased connectivity with hippocampus and cerebellum. Numerous nonhuman primate studies have demonstrated that the PRC and PHC provide nearly two-thirds of the cortical input to the ERC. The anterolateral aspects of the ERC receive highly integrated visual object information from occipito-temporal visual object processing pathway via the PRC^7^,^35^, whereas the posteromedial aspects of the ERC receive visuospatial information primarily from the parieto-medial temporal visuospatial pathway via the PHC^36^. Focusing on the electrophysiological properties of neurons, numerous studies have indicated that the ERC neurons may provide key information about how episodic memories are formed in downstream hippocampus^52^,^53^. Therefore, evidences from previous studies as well as our findings suggest that the disconnection of the ERC and HIP is associated with episodic memory dysfunction^54^,^55^. Furthermore, it is interesting to note that the disconnection of the ERC and HIP accompanied with the disconnection of ERC and cerebellum, which is line with our previous studies that cerebellum deficits in aMCI were associated with the changes in the hippocampus^56^, suggesting the cerebellum deficits in aMCI may have physiological meaning^57^,^58^. It is consistently suggested that the impairment on structural and functional levels in the cerebellum in elderly aMCI may be related to cognitive dysfunction. Therefore, this result adds to our understanding of cerebellum by noting that this area is likely to be a connectional “hub” to the hippocampus by way of the ERC.

Our findings complement findings of functional heterogeneity along the longitudinal axis of the HIP in both animals and humans. The present study showed the decreased connectivities between right aHIP and visual cortex (right fusiform gyrus), between mHIP and parietal cortex (precuneus), middle temporal gyrus, cerebellum, and visual cortex, between right pHIP and visual cortex in aMCI. These results suggest that aMCI present differentially abnormal connectivity patterns along the anterior to posterior axis of HIP. The previous studies have indicated that the fusiform gyrus, which is located on the ventromedial surface of the temporal and occipital lobes^59^, is a key structure involved in facial cognition^60^,^61^,^62^. aMCI patients showed altered FC of the fusiform gyrus^61^ and widespread changes in fusiform connectivity during the performance of a face-matching task^60^. Previous studies have reported that the anterior hippocampus may be preferentially involved in memory for faces^63^. Therefore, our findings suggest that aMCI subjects present visual facial cognition deficits^64^, which is associated with early clinical symptom of aMCI. The reduced connectivity between fusiform gyrus and HIP may be due to the presence of cholinergic lesions in aMCI^65^; specifically, it has been reported that cholinergic deficits exist in the primary visual cortex in mild AD^66^. In this context, reduction connectivity between the precuneus and HIP is particularly noteworthy, since the precuneus plays a key role in how intrinsic activity is mediated throughout the DMN^67^. Previous studies have also reported a precuneus connectivity reduction in the DMN^49^,^50^,^68^ and reduced FC between the precuneus and the MTL in aMCI^20^, which is a potentially valuable indicator of conversion from aMCI to AD.

### Behavioral significance of the altered functional connectivity of MTL

The relationship between the decreased FC and the neuropsychological performance supported the notion that the disrupted functional neurocircuitry was the basis of cognitive impairment in aMCI (Fig. 4), suggesting that the functional changes of the right HIP-ERC-PRC-temporal pathway lead to the impairment of episodic memory. Several studies demonstrate that the HIP binds multisensory object and spatial, contextual, and associational information together to represent our semantic and episodic memories^19^,^69^. These memories bind both spatial or context information from the dorsal stream, transmitted via PHC–ERC connections^36^, together with object information received via PRC–ERC connections^35^. Taken together, these results indicate that the disruption of the occipito-temporal visual object processing pathway may contribute to the impairment of episodic memory in aMCI^7^,^25^.

### Potential significance of the relationships between the aging process and increased functional connectivity in aMCI

Interestingly, the present study demonstrated that the relationships between the increased FC strength and age were quadratic, namely reverse U-shaped curve (Fig. 4A,C). This finding is consistent with models of brain compensation and plasticity in ageing, suggesting that the brains of MCI remain highly plastic and interventions can be provide to promote brain plasticity^70^. Our results suggest that there may be a phase of increased FC in MTL at the clinical stage of aMCI, that is, with the aging process, the MTL connectivity may lose some compensation and add some more disruption due to the pathological changes. Furthermore, the peak of the inverse U-shaped curve was estimated to locate at around 70 years old, consistent with a series of findings demonstrating that the compensatory mechanism in aMCI may play the greatest role at their 70s when gradually starts to collapse over 70s. The upgoing phase of the curve before 70s represents minimal pathological stage while the downgoing phase over 70s indicates the gradual destruction of compensatory mechanism related to pathological progression in aMCI^71^. Therefore, this finding also indicate that care must be taken to use rs-fMRI on an individual basis as a physiologic imaging biomarker for diagnostic attempts, depending on the disease stage. Future studies should focus on the relationship between this U-shaped functional curve and other anatomical (e.g. atrophy) or metabolic (e.g. Aβ deposition) markers. Further, a combination of clinical (e.g., MMSE scores), neuropsychologic (e.g., memory tasks), anatomic, and metabolic measures may be used to assist in the determination of where an individual is along the inverse U-shaped functional curve of MTL.

## Limitations

The resting data of this study were smoothed with a Gaussian kernel of 4 × 4 × 4 mm^3^, so each ROI would have included mixed signals from adjacent regions. To assess the effect degree of the resting connectivity patterns by the choice of smoothing, we compared the differences of these results from the different smoothing sizes (e.g. 2 × 2 × 2 mm^3^, 4 × 4 × 4 mm^3^, and 6 × 6 × 6 mm^3^) and no significant differences were found.

## Conclusion

The current study provides novel insights into the heterogeneous nature of its large-scale connectivity in MTL subregions underlying the memory deficits in aMCI. It further suggests that the functional changes in the right HIP-ERC-PRC-temporal circuitry may be an early indicator for early stage changes and progression of this disease.

## Materials and Methods

### Ethics statement

This study was approved by the responsible Human Participants Ethics Committee of the Affiliated ZhongDa Hospital, Southeast University. All participants were assessed at the Affiliated ZhongDa Hospital, Southeast University, and written informed consent was obtained from all of the participants and the methods were carried out in accordance with the approved guidelines.

### Participants

The present study initially recruited 222 elderly individuals (all of whom were Chinese Han and right-handed), including 87 aMCI, and 135 HC, through normal community health screening, newspaper advertisement, and hospital outpatient service. Due to motion artifacts, of these, 6 HC subjects and 2 aMCI subjects were excluded. The remaining 129 HC and 85 aMCI subjects were eligible and entered further analysis. The inclusion and exclusion criteria (see details in *SI Methods*) used to choose subjects can be found in our previously published studies^56^.

### Neuropsychological assessments

All subjects underwent a standardized clinical interview and comprehensive neuropsychological assessments that were performed by neuropsychologists (Dr. Shu, Wang, and Liu). The details regarding the neuropsychological assessments are provided in *SI Methods*.

### MRI data acquisition

MRI images were acquired using a 3.0 Tesla Trio Siemens scanner (Siemens, Erlangen, Germany) with a 12-channel head-coil at ZhongDa Hospital Affiliated to Southeast University. Details on image acquisition parameters are provided in *SI Methods*.

### Image preprocessing

Data analyses were conducted with Matlab (Math Works Inc., Natick, MA, USA) and SPM8 (available at: http://www.fil.ion.ucl.ac.uk/spm). Briefly, this preprocessing included removal of the first ten volumes, slice timing correction and head motion correction^72^,^73^. To spatially normalize the rs-fcMRI data, the T1-weighted images were used to register the functional data to their corresponding anatomical image, and the resulting aligned T1 dataset was transformed into Montreal Neurological Institute (MNI) space^74^. To improve the coregistration of the rs-fcMRI data, a custom T1 template was built by averaging the normalized anatomical images across all subjects^75^. Finally, the normalized functional images were created by applying the transformation of the T1 images to the customized T1 template. Notably, such a custom template-based registration procedure could reduce the inaccuracy of the spatial normalization of functional volumes due to GM atrophy in aMCI and HC. Functional images were resampled to 2 × 2 × 2 mm^3^ voxels and spatially smoothed using a 4-mm full-width half-maximum (FWHM) Gaussian kernel. Linear detrending and temporal band-pass filtering (0.01–0.08 Hz) were applied to reduce the effect of low-frequency drifts and high-frequency physiological noise. Finally, several nuisance variables, including six head motion parameters, global mean signal^76^,^77^, cerebrospinal fluid (CSF), and WM signal were removed by multiple linear regression analysis.

### Quality assurance (QA)

#### Assessment of susceptibility artifacts

Previous studies have indicated that brain areas in the MTL directly above the petrous bone especially tend to signal loss^17^,^78^. To assess the effects of susceptibility artifacts in our data, the signal-to-noise ratio (SNR) was computed for each voxel by averaging the signal intensity across all the target-atlas normalized BOLD runs and dividing it by the SD over time^79^. To avoid variability in EPI timeseries cause of susceptibility artifact, all ROIs were thresholded to exclude voxels with mean signal in the intensity-normalized EPI timeseries below 3000, corresponding approximately to a SNR of 20^18^,^80^. Thresholds resulted in the rejection of no more than 5% of voxels in any ROI. Then these thresholded ROIs were further used to perform FC analyses.

#### Gray matter loss effect

As numerous studies have indicated significant GM atrophy in aMCI^56^,^81^, these differences on FCs of the MTL subregions in our study might be driven by the anatomical differences between groups. To clarify this issue, we performed the general linear model (GLM) analysis to examine the between-group differences on the FCs for each MTL subregion with GM volume as an additional covariate^30^,^82^. Briefly, the resultant GM volume map of each individual was first estimated from the normalized T1 images using Voxel-Based Morphometry (VBM8 toolbox, http://dbm.neuro.uni-jena.de/vbm). Then, a two-sample *t* test was performed to determine whether the GM was atrophied in aMCI (Fig. S4), controlling for age, gender, and years of education. Details on VBM analysis are provided in *SI Methods*.

#### Head motion effect

To minimize the influence of head motion both at the individual and at the group levels, three approaches were employed in QA measures. First, the head motion effects were regressed out, which were calculated as the root mean squared (rms) head displacement or rotation (in mm or °) derived from the motion-correction procedure^76^. Second, a ‘scrubbing’ procedure was carried out to scrub frames (volumes) with excessively high whole-brain rms signal change over time in the preprocessed rs-fcMRI data for each individual^47^,^72^,^73^. We discarded a fraction of <7% in each group (no significant differences on fraction of frames removed were found). Overall, 2 HC and 2 aMCI had a large proportion of high-noise frames (>20% frames identified as contaminated) and were therefore excluded from the analysis. Third, additional QA measures included rms head displacement or rotation (in mm or °) and the voxel-wise time series standard deviation (SD) averaged over the whole brain^73^,^83^. We referred to a prior study^84^ to empirically determine the exclusion criteria for QA measure with the objective of achieving QA parameter distribution equivalence between groups while maximizing the number of included subjects. Overall, 4 HC subjects with a mean preprocessed rs-fcMRI signal 2.5% SD (after nuisance regression) or rms movement or rotation exceeding 1.5 mm or 1.5° and mean frame-to-frame rms movement or rotation more than 0.15 mm or 0.1° were also excluded. The QA parameters before and after the removal of contaminated frames are shown in Table S2. No significant differences between groups were observed in QA parameters (Table S2).

#### Definition of MTL seed ROIs

**Figure 1:**
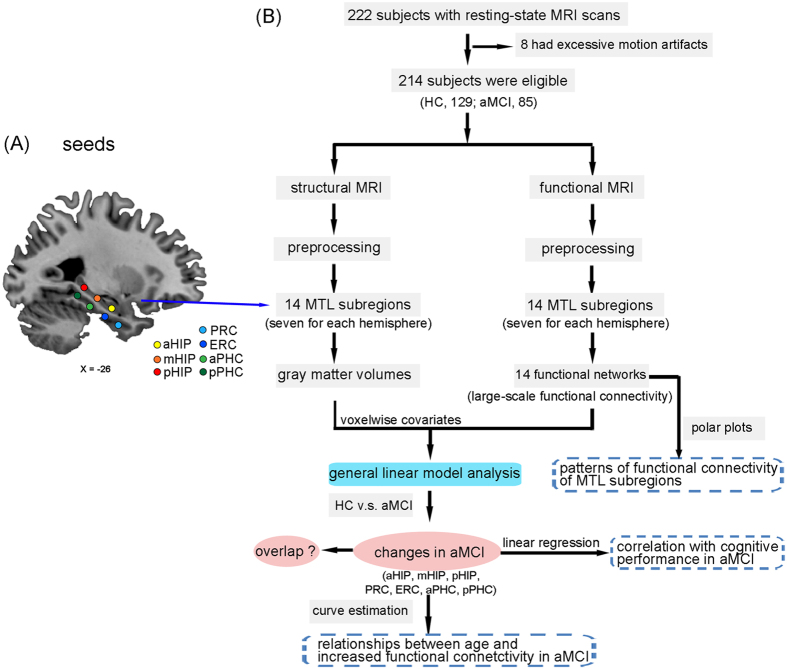
Seven seed regions of interest in the left hemisphere and schematic of data analysis pipeline. (**A**) A representative sagittal view of the brain depicts seven seed locations along the longitudinal axis of the hippocampus and the parahippocampal gyrus. Seeds were superimposed on a high-resolution T1-weighted brain template in stereotaxic Montreal Neurological Institute (MNI) space. (**B**) Schematic of data analysis pipeline. Regional mean fMRI time series were estimated by a custom T1 template, which was built by averaging the normalized anatomical images across all subjects. Gray matter volumes were also estimated for each of the same set of 14 MTL subregions used to parcellate the fMRI data. The general linear model analyses compared aMCI group to HC subjects on all neuroimaging markers; the multiple linear regression was used to examine relationships between the functional connectivity and cognitive performance scores; and the curve estimation was used to examine the relationships between increased functional connectivity and age in aMCI patients. Futhermore, schematic polar plots were quantitatively used to summarize overall functional connectivity patterns of each seed with target regions throughout the whole-brain. Abbreviations: aHIP, anterior hippocampus; mHIP, middle hippocampus; pHIP, posterior hippocampus; PRC, perirhinal cortex; ERC, entorhinal cortex; aPHC, anterior parahippocampal cortex; pPHC, posterior parahippocampal cortex; MRI, magnetic resonance imaging; MTL, medial temporal lobe; HC, healthy controls; aMCI, amnestic mild cognitive impairment.

**Figure 2:**
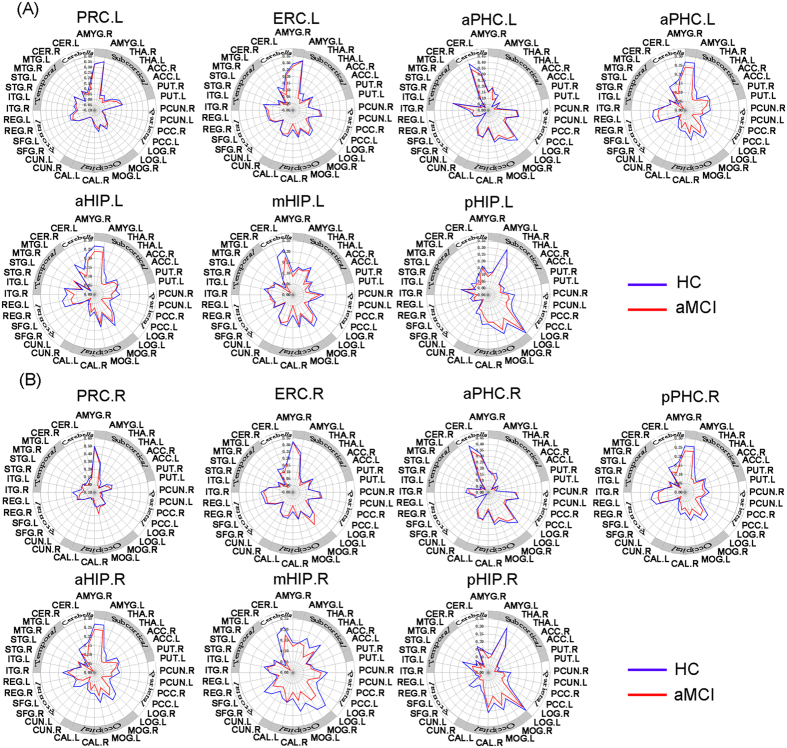
Heterogeneous functional connectivity associated with distinct parahippocampal and hippocampal seeds in HC and aMCI subjects ((**A**) left hemisphere seeds; (**B**) right hemisphere seeds). Schematic polar plot depicts connectivity patterns of four parahippocampal and three hippocampal seeds with target ROIs distributed across the whole brain. The concentric circles depict parameter estimates representing the connectivity strength. Note that the data of functional connectivity are extracted from the only brain regions corresponding to Fig. S1. Abbreviation: AMYG, amygdala; THA, thalamus; ACC, anterior cingulate gyrus; PUT, putamen; PCUN, precuneus; PCC, posterior cingulate cortex; LOG, inferior occipital gyrus; MOG, middle occipital gyrus; CAL, calcarine gyrus; CUN, cuneus; SFG, superior frontal gyrus; REG, rectus gyrus; ITG, inferior temporal gyrus; STG, superior temporal gyrus; MTG, middle temporal gyrus; CER, cerebelum.

**Figure 3:**
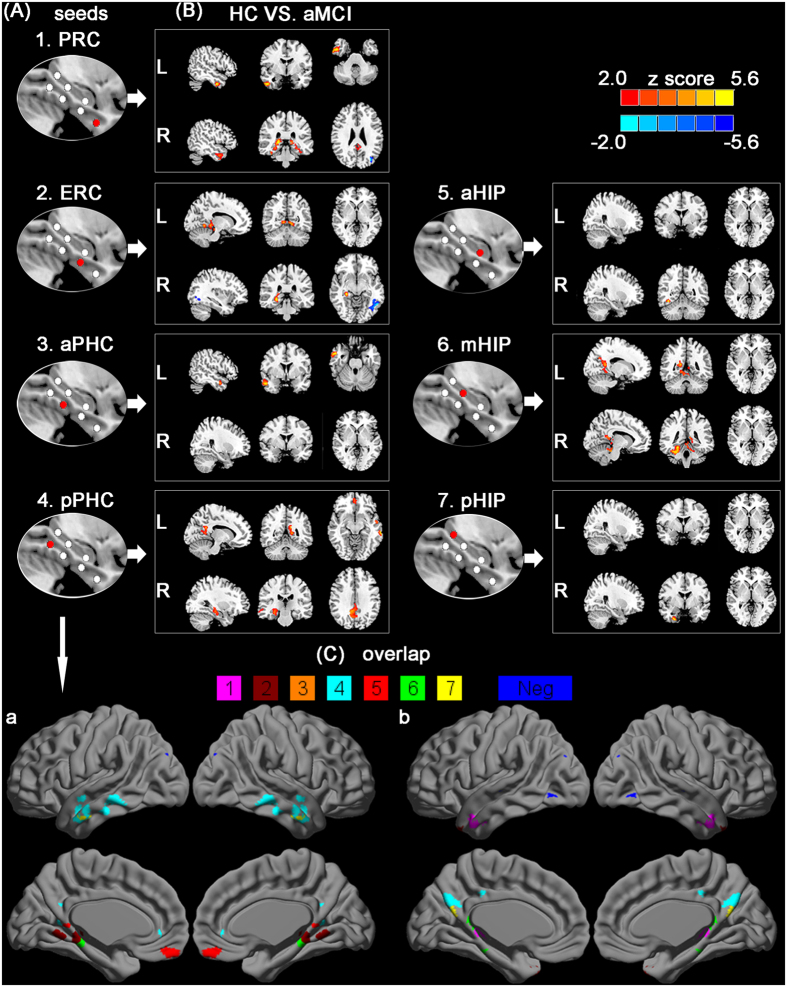
Resting-state functional connectivity patterns between group maps of the distinct MTL subregions. (**A**) First column shows the subregions defined in MTL. (**B**) Last column shows the between-group statistical maps, with statistical threshold set at *p*_corrected_ < 0.01, corrected by AlphaSim. Warm and blue colors indicate decreased and increased functional connectivity in aMCI subjects compared to healthy controls. Color bar is presented with z score, respectively. (**C**) The spatial overlap in left (a) and right (b) parahippocampal and hippocampal seeds shows all brain regions of disrupted functional connectivity in aMCI subjects compared to healthy controls. The color bar (1–7 colors) indicates disrupted connectivity maps derived from each seed. Blue color shows these regions of increased functional connectivity in aMCI subjects compared to healthy controls. Notes: aHIP, anterior hippocampus; mHIP, middle hippocampus; pHIP, posterior hippocampus; PRC, perirhinal cortex; ERC, entorhinal cortex; aPHC, anterior parahippocampal cortex; pPHC, posterior parahippocampal cortex; L, the left MTL subregional networks; R, the right MTL subregional networks.

**Figure 4:**
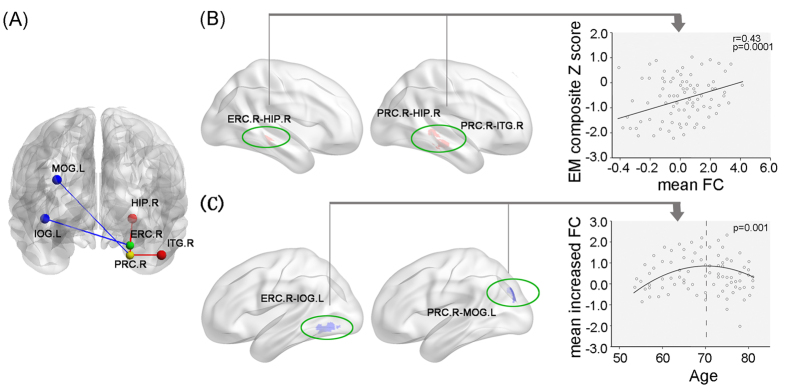
Behavioral significance of disrupted MTL network connectivity in aMCI patients. (**A**) Schematic plot indicates the relationships between increased and decreased functional connectivity in aMCI patients compared to healthy controls. Blue color shows increased functional connectivity, and red color shows decreased functional connectivity. (**B**) Scattergrams represent he correlations between clinical variables and resting-state FC of MTL subregions in aMCI patients. (**C**) The reverse u-shaped curve depicts the relationship between age and mean Z values of increased functional connectivity in aMCI patients compared to healthy controls. Abbreviations: FC, functional connectivity; EM, episodic memory; ITG, inferior temporal gyrus; IOG, inferior occipital gyrus; MOG, middle occipital gyrus; HIP, hippocampus; PRC, perirhinal cortex; ERC, entorhinal cortex; L, left; R, right.

**Figure 5:**
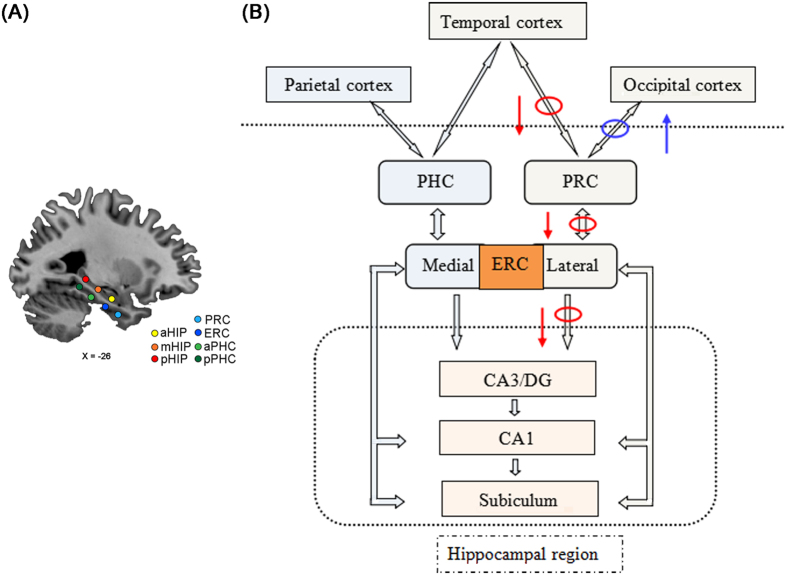
Schematic drawing of medial temporal lobe (**A**) plus the components of the medial temporal lobe memory system believed to be important in the transition from perception to memory (**B**). The networks in cortex show representations of occipito-temporal visual object processing pathway (the “what” stream) and parieto- temporal visuospatial pathway (the “where” stream) for the formation of memory. Red circle and arrow complex representing disconnected neural pathway associated the disruption of episodic memory in the aMCI patients. Blue circle and arrow complex representing compensated neural pathway in the aMCI patients.

This study created two parallel arrays of four-millimeter spherical seeds along the longitudinal axis of the PHG and HIP respectively (Fig. 1A). Specifically, the four seeds appropriately located at the PRC (i.e. PRC), the transition area of ERC and posterior PRC region (i.e. ERC), the anterior and posterior PHC (i.e. aPHC, pPHC). Moreover, three other seeds appropriately located at the head (anterior), body (middle) and tail (posterior) of the HIP (i.e. aHIP, mHIP, pHIP) (Table 4 for the details of MNI seed coordinates). The precise locations of these seeds were defined based on previous studies in animals and humans about distinct anatomical and functional profiles of MTL subdivisions^11^,^17^,^18^,^19^, and functional heterogeneity along the HIP axis^85^,^86^,^87^. All specific ROIs were created by drawing 4-mm spheres, of which central coordinates were determined and defined in the MNI stereotaxic space according to the well-established MNI seed coordinates that originally represented the MTL subdivisions as described in previously published studies^11^,^17^,^18^,^26^,^88^ (Table 4 for the details of MNI seed coordinates). To ensure the accuracy and correspondence of those ROIs across different subjects, all ROIs were used to extract an averaged hemodynamic timeseries for each location, and for each subject, by applying each ROI mask to the preprocessed timeseries, and averaging across all voxels within the ROI.

#### Functional connectivity analyses

The average time courses for all voxels within each MTL subregion were extracted as the reference time course, separately, and voxelwise cross-correlation analysis was then carried out between the averaged time courses of all voxels within the seed region and the whole brain within the GM mask. The GM mask was created by thresholding (a probability threshold of 0.2) the GM probability map obtained from all subjects in this study. A Fisher’s z-transform was then applied to improve the normality of the correlation coefficients.

### Statistical analysis

#### Demographic and neuropsychological data

The statistical analyses were performed using SPSS 20.0 software (SPSS Inc., Chicago, IL, USA). The two-independent samples *t* test and chi-square tests (applied only in the comparisons according to gender) were used to test the differences in demographic data and neuropsychological performances between aMCI and HC (*p* < 0.05).

#### Group-level intrinsic connectivity analysis

To highlight the FC patterns of MTL subregions at a group level, the spatial maps of FC in each group were submitted to a random-effect analysis using one-sample *t*-tests with a stringently threshold of *p* < 0.01 using family-wise error (FWE) correction together with a cluster extent k > 100 voxels (800 mm^3^) to reveal regions most robustly correlated with each seed. Only clusters within the GM mask were retained. To avoid ambiguous biological interpretations related to apparently negative connectivity resulting from correction for global signal changes^77^, only positive FC was used in this study.

To assess the between-group differences of the FCs of each MTL subregion, we used GLM analysis (“FCs” as dependent variable, “group” as independent variable) with age, gender, years of education, and GM volume treated as covariates. A statistical threshold of *p* (uncorrected) <0.005 and cluster extent k > 80 voxels (640 mm^3^) was used to achieve a corrected statistical significance of *p* < 0.05, determined by Monte-Carlo simulation (see program AlphaSim by D. Ward).

To assess the potential overlap between the between-group difference connectivity maps derived from the seven seeds of interest, these between-group difference maps from GLM analysis were thresholded as indicated above, binarized and summed, generating a so-called “overlapping” map.

To characterize abnormal FC patterns of MTL seeds to specific target brain ROIs, schematic polar plots were used to describe FC patterns of each seed with target regions throughout the whole-brain. Details on definition of target ROIs are provided in *SI Methods*. Analysis of variance (ANOVA) was used to test the difference of FC patterns of MTL seeds between aMCI and HC (*p* < 0.05).

#### Correlations between the altered MTL subregional connectivity and neuropsychological performance

To assess the links between altered FC and neuropsychological performance, we extracted the averaged FC strengths of the regions showing altered MTL subregional connectivity. The multiple linear regression model analysis was used to examine the relationships between the extracted FC strengths and the neuropsychological performance in each group. To increase statistical power by reducing random variability, as previously described^82^,^89^, we composited the neuropsychological tests into 4 cognitive domains and transformed the raw scores into 4 composite Z scores (see details in *SI Methods*) (*p* < 0.05, Bonferroni-corrected).

#### Independent associations between the aging process and increased FC

To investigate the links between the age and increased FC in the whole-brain in aMCI due to HC, the curve estimation was used to examine the relationships between FC strength (mean Z values of increased FC in the whole-brain) and age in each group (*p* < 0.05, Bonferroni-corrected).

## Additional Information

**How to cite this article**: Chen, J. *et al.* Differential contributions of subregions of medial temporal lobe to memory system in amnestic mild cognitive impairment: insights from fMRI study. *Sci. Rep.* **6**, 26148; doi: 10.1038/srep26148 (2016).

## Supplementary Material

Supplementary Information

## Figures and Tables

**Table 1:** Demographics and clinical measures of aMCI patients and healthy control subjects.

Items	HC	aMCI	*t* values(χ^2^)	*p*values
	n = 129	n = 85		
Age (years)	68.43(6.59)	69.48(7.52)	−1.085	0.279
Gender (male/female)	64/65	46/39	0.416	0.519
Education level (years)	12.39(3.04)	11.84(3.22)	1.271	0.205
MMSE scores	28.22(1.43)	26.25(2.65)	7.035	0.000^*^
MDRS_2	137.92(3.57)	131.29(6.85)	9.252	0.000^*^
HAMD scores	1.63(2.39)	1.81(3.11)	−0.481	0.631
Composite Z scores of each cognitive domain				
Episodic memory	0.50(0.50)	−0.75(0.71)	15.266	0.000^*^
information processing speed	−0.12(0.35)	0.19(0.59)	−4.78	0.000^*^
Executive function	0.04(0.35)	−0.07(0.36)	2.292	0.000^*^
Visuospatial function	0.32(0.66)	−0.04(0.35)	7.768	0.000^*^

Notes: Values are expressed as the mean (standard deviation, SD). Abbreviations: HC, healthy controls; aMCI, amnestic mild cognitive impairment; MMSE, Mini mental state exam; MDRS-2, Mattis Dementia Rating Scale-2; HAMD, Hamilton Depression Scale. ^*^Significant differences are found between aMCI and HC subjects. *P* values are obtained by *t* test except for gender (chi square test). The performances of MMSE and MDRS-2 are presented as raw scores. The level of each cognitive domain is denoted by the composite Z scores. Raw scores and corresponding Z scores of individual neuropsychological tests are shown in Supplementary Table1.

**Table 2:** Comparisons of functional connectivity of the parahippocampal seeds between aMCI patients and HC subjects.

Brain region	Peak MNI coordinate			Peak *Z* value	Cluster size (mm^3^)
	x	y	z		
The left PHG subregional networks					
(1) PRC (HC > aMCI)					
** **R Inferior temporal gyrus	52	2	−32	2.89	723
(2) ERC (HC > aMCI)					
L Cerebelum_4_5	−8	−56	−2	3.31	752
R Cerebelum_4_5	10	−52	−4	3.92	721
(3) aPHC (HC > aMCI)					
R Middle temporal gyrus	52	2	−26	3.67	1336
(4) pPHC (HC > aMCI)					
L Inferior temporal gyrus	−62	−36	−14	3.30	960
L Middle temporal gyrus	−66	−30	−12	3.43	1560
R Middle temporal gyrus	48	2	−30	3.60	1364
The right PHG subregional networks					
(1) PRC (HC > aMCI)					
R Hippocampus	20	−32	2	4.43	792
R Inferior temporal gyrus	52	−4	−36	3.62	984
L Thalamus	−14	−34	2	3.57	624
(HC < aMCI)					
L Middle occipital gyrus	−34	−78	26	−3.40	636
(2) ERC (HC > aMCI)					
R Hippocampus	28	−30	−6	3.22	684
(HC < aMCI)					
L Inferior occipital gyrus	−44	−66	−6	−3.37	682
(3) aPHC (HC > aMCI)					
none					
(4) pPHC (HC > aMCI)					
** **R Angular gyrus	42	−64	36	3.66	680
** **L Cuneus	−20	−58	26	3.90	616
** **L Precuneus	0	−64	28	3.09	776
** **R Precuneus	10	−50	34	3.16	1744
** **R Middle temporal gyrus	60	−32	−8	3.41	784

Abbreviations: HC, healthy controls; aMCI, amnestic mild cognitive impairment; PRC, anterior perirhinal cortex; ERC, entorhinal cortex; aPHC, anterior parahippocampal cortex; pPHC, posterior parahippocampal cortex.

**Table 3:** Comparisons of functional connectivity of the hippocampus subregional seeds between aMCI patients and HC subjects.

Brain region	Peak MNI coordinate			Peak *Z* value	Cluster size (mm^3^)
	x	y	z		
The left HIP subregional networks					
(1) aHIP (HC > aMCI)					
none					
(2) mHIP (HC > aMCI)					
** **R Precuneus	16	−50	14	3.23	800
** **R Middle temporal gyrus	60	4	−30	3.80	1336
(3) pHIP (HC > aMCI)					
none					
The right HIP subregional networks					
(1) aHIP (HC > aMCI)					
** **R Fusiform gyrus	38	−52	−14	3.79	872
(2) mHIP (HC > aMCI)					
** **R Cerebelum_4_5	24	−40	−24	3.62	1720
** **R Fusiform gyrus	24	−40	−16	3.75	1456
(3) pHIP (HC > aMCI)					
** **R Fusiform gyrus	30	−2	−40	3.29	744

Abbreviations: HC, healthy controls; aMCI, amnestic mild cognitive impairment; aHIP, anterior hippocampus; mHIP, middle hippocampus; pHIP, posterior hippocampus.

**Table 4:** Regions, MNI coordinates, and sample of references supporting choice of seven seed regions of interest.

ROI	MNI coordinates			Functional references	Anatomical references
	x	y	z		
PRC	(±) 26	−4	−36	Kahn *et al.*^17^; Libby *et al.*^18^; Lacy and Stark^26^	Colombo *et al.*^85^; Maguire *et al.*^86^; Strange and Dolan 2006; Poppenk and Moscovitch^87^; Staresina *et al.*^11^; Kivisaari *et al.*^19^
ERC	(±) 26	−16	−28	Kahn *et al.* 17; Lacy and Stark^26^	
aPHC	(±) 26	−30	−20	Kahn *et al.*^17^; Libby *et al.*^18^; Lacy and Stark^26^	
pPHC	(±) 26	−40	−12	Kahn *et al.*^17^; Libby *et al.*^18^; Lacy and Stark^26^	
aHIP	(±) 24	−14	−20	Kahn *et al.*^17^; Libby *et al.*^18^; Lacy and Stark^26^; Yassa *et al.*^88^	
mHIP	(±) 26	−26	−12	Kahn *et al.*^17^; Lacy and Stark^26^; Yassa *et al.*^88^	
pHIP	(±) 26	−34	−4	Kahn *et al.*^17^; Libby *et al.*^18^; Lacy and Stark^26^; Yassa *et al.*^88^	

References included contain ROIs that are within the expanded seed ROIs used in this study. Abbreviations: PRC, anterior perirhinal cortex; ERC/pPRC, posterior perirhinal cortex proximate to entorhinal cortex; aPHC, anterior parahippocampal cortex; pPHC, posterior parahippocampal cortex; aHIP, anterior hippocampus; mHIP, middle hippocampus; pHIP, posterior hippocampus. Coordinates for seeds in the right and left hemispheres are defined in the MNI stereotaxic space.
