# Supplementary material for: Differential contributions of subregions of medial temporal lobe to memory system in amnestic mild cognitive impairment: insights from fMRI study
Source: Sci Rep. 2016 May 17;6:26148. doi: 10.1038/srep26148 (PMC4868964; doi:10.1038/srep26148)
Supplement: Supplementary Information [file srep26148-s1.pdf]

Differential contributions of subregions of medial temporal lobe to memory system in amnestic mild cognitive impairment: insights from fMRI study

Jiu Chen[1, *], Xujun Duan[2, *], Hao Shu[1], Zan Wang[1], Zhiliang Long[2], Duan Liu[1], Wenxiang Liao[1], Yongmei Shi[1], Huafu Chen [2, §] & Zhijun Zhang [1, 3§]

[1] Department of Neurology, Affiliated ZhongDa Hospital, School of Medicine, Southeast University, Nanjing, Jiangsu 210009, PR China

[2] Key Laboratory for NeuroInformation of Ministry of Education, School of Life Science and Technology, University of Electronic Science and Technology of China, Chengdu 610054, PR China

[3] Department of Psychology, Xinxiang Medical University, Xinxiang, Henan 453003, China

[*] These authors contributed equally to this work.

[§]Corresponding author:

Zhijun Zhang, Department of Neurology, Affiliated ZhongDa Hospital, Medical School, Southeast University, No. 87 Dingjiaqiao Road, Nanjing, Jiangsu, China, 210009, E-mail: janemengzhang@vip.163.com;

and

Huafu Chen, Key Laboratory for NeuroInformation of Ministry of Education, School of Life Science and Technology, University of Electronic Science and Technology of China, Chengdu 610054, PR China, E-mail: chenhf@uestc.edu.cn.

Supplementary Information

SI methods

Participants:

To be considered for inclusion, participants had to have had functional connectivity and structural MRI performed in ZhongDa Hospital Affiliated to Southeast University on a Neuroscience Imaging Center 3-T scanner.

All aMCI subjects met the diagnostic criteria proposed by Petersen and colleagues [1] and the revised consensus criteria of the International Working Group on aMCI [2], including (1) subjective memory impairment corroborated by the subject and an informant, (2) objective memory performance documented according to an Auditory Verbal Memory Test-delayed recall score that was within ≤ 1.5 SD of age- and education-adjusted norms (the cutoff was ≤ 4 correct responses on 12 items for ≥ 8 years of education), (3) normal general cognitive function as evaluated by a MMSE of 24 or higher, (4) a Clinical Dementia Rating of 0.5, with at least a 0.5 in the memory domain, (5) no or minimal impairment in daily living activities, and (6) absence of dementia, symptoms that were not sufficient to meet the criteria of the National Institute of Neurological and Communicative Disorders and Stroke or the AD and Related Disorders Association criteria for AD. Exclusion criteria were as follows: (1) a past history of known stroke (modified Hachinski score of > 4), alcoholism, head injury, Parkinson's disease, epilepsy, major depression (excluded by Self-Rating Depression Scale), or other neurological or psychiatric illness (excluded by clinical assessment and case history), (2) major medical illness (e.g., cancer, anemia, and thyroid dysfunction), (3) severe visual or hearing loss, and (4) T2-weighted MRI showing major white matter (WM) changes, infarction, or other lesions (two experienced radiologists analyzed the scans).

The HC subjects were required to have a clinical dementia rating of 0, an MMSE score of ≥ 26, and a delayed recall score of > 4 for those with ≥ 8 years of education. These participants were matched by subject to aMCI subjects. All subjects underwent a standardized clinical interview including demographic inventory, medical history and neurological and mental status examination. Group-specific information for the subjects is provided in Table 1.

Neuropsychological assessments

As our previously published described [3,4], all subjects underwent a standardized clinical interview and comprehensive neuropsychological assessments that were performed by neuropsychologists (Dr. Shu, Wang, and Liu), including the Mini-Mental State Examination (MMSE), Mattis Dementia Rating Scale-2 (MDRS-2), Auditory Verbal Learning Test – immediate recall (AVLT-IR), Auditory Verbal Learning Test – 5-min delayed recall (AVLT-5-min-DR), Auditory Verbal Learning Test – 20-min delayed recall (AVLT-20-min-DR), Logical Memory Test-immediate recall (LMT-IR), Logical Memory Test – 20-min delayed recall (LMT-20-min-DR), Rey-Osterrieth Complex Figure Test (ROCFT), Rey-Osterrieth Complex Figure Test – 20-min delayed recall (ROCFT-20min-DR), Trail-Making Tests A and B (TMT-A and B), Digital Symbol Substitution Test (DSST), Digit Span Test (DST), Stroop Color and Word Test A, B, and C, Verbal Fluency Test (VFT), Semantic Similarity (Similarity) test, and Clock Drawing Test (CDT). These tests were used to evaluate general cognitive function, episodic memory, information processing speed, executive function,

perceptual speed, working memory, and visuo-spatial function, respectively.

Image Acquisition, and Analysis. *Functional connectivity data: Acquisition.*

MRI images were acquired using a 3.0 Tesla Trio Siemens scanner (Siemens, Erlangen, Germany) with a 12-channel head-coil at Zhongda Hospital Affiliated to Southeast University. Resting-state functional images including 240 volumes were obtained using a gradient-recalled echo-planar imaging (GRE-EPI) sequence, with repetition time (TR) = 2000 ms, echo time (TE) = 25 ms, flip angle (FA) = 90°, acquisition matrix = 64 × 64, field of view (FOV) = 240 mm × 240 mm, thickness = 4.0 mm, gap = 0 mm, number of slices = 36, and voxel size = 3.75 × 3.75 × 4 mm^3. High-resolution T1-weighted axial images covering the whole brain were acquired by 3D magnetization prepared rapid gradient echo (MPRAGE) sequence as described below: TR = 1900 ms, TE = 2.48 ms; FA = 9°, acquisition matrix = 256 × 256, FOV = 250 × 250 mm, thickness = 1.0 mm, gap = 0 mm, number of slices = 176, and voxel size = 1 × 1 × 1 mm^3. Additionally, routine axial T2-weighted image were acquired to rule out subjects with major WM changes, cerebral infarction or other lesions using flair sequence as described below: TR = 8400 ms, TE = 94 ms, FA= 150°, acquisition matrix = 256×256, FOV = 230×230 mm, thickness = 5.0 mm, gap = 0 mm, and number of slices = 20.

Voxelwise-based gray matter volume correction

Voxel-based morphometry (VBM) analysis was performed using VBM8 toolbox in SPM8 (http://www.fil.ion.ucl.ac.uk/spm/). The individual T1-weighted images were segmented into gray matter (GM), WM and CSF, and then normalized to the MNI space and resampled to 2×2×2 mm^3 cubic voxel. Finally, the voxelwise values were smoothed with a Gaussian kernel of 4×4×4 mm (full-width half-maximum FWHM) as the functional images. The resultant GM volume maps were input as voxelwise covariates in further analysis to ensure the difference of functional connectivity (FC) not driven by the anatomical difference between groups.

Statistical analysis

Group-level intrinsic connectivity analysis

Repeated-measure ANOVA was used to test the difference of FC patterns along the anterior-posterior PHG and HIP axis in HC subjects. A post-hoc Student's t-test for each two pairs of seeds was carried out to further investigate differences as any statistical significance for ANOVA. $p < 0.05$ was required for statistical significance.

The relationships between the altered MTL subregional pattern and neuropsychological performance

To increase statistical power by reducing random variability, this study composited the neuropsychological tests into 4 cognitive domains and transformed the raw scores into 4 composite Z scores, as previously described [3,4]. First, for each neuropsychological test, the individual raw scores were transformed to Z scores, according to the mean and standard deviation of the scores for all subjects. Notably, for tests measured by timing, including TMT-A, TMT-B, Stroop A, Stroop B, and Stroop C, the raw scores were defined as the reciprocal of the time required for the test. Then,

each cognitive domain's composite Z score was determined by averaging the Z scores related to the tests. We divided these tests into 4 cognitive domains: episodic memory (3 tests, including AVLT-20-min DR, LMT-20-min DR, and CFT-20-min DR), information processing speed (4 tests, including DSST, TMT-A, Stroop A, and Stroop B), visuospatial function (2 tests, including CFT and CDT), and executive function (5 tests, including VFT, DST-backward, TMT-B, Stroop C, and Similarity). All statistical procedures utilized SPSS 17.0 software (SPSS Inc., Chicago, IL, USA). Bonferroni correction for multiple comparisons (24 altered seed-target pairs×4 composite cognitive tests) was performed at a significance level of $p < 0.0005$ ($p = 0.05/96$).

SI Results

Different large-scale FC patterns among four PHG seeds and three HIP seeds

Schematic polar plots demonstrated that there were different FC patterns among four PHG seeds **(Fig. S1, Fig. S2, Fig. S3)**. The aPHC network showed much stronger connectivity with widely distributed brain regions in cerebella – parietal – occipital system (PCUN, MOG, CAL, and CER regions) compared with other three seeds, whereas the PRC, ERC, and pPHC networks showed the similar FC patterns. The PRC network showed much stronger connectivity with the AMYG, PUT, MOG, and REG regions. The ERC network showed much stronger connectivity with the AMYG, PCUN, and ITG regions. The pPHC network showed much stronger connectivity with the AMYG, PUT, LOG, and REG regions.

Schematic polar plots demonstrated that there were different FC patterns among anterior, middle and posterior HIP **(Fig. S1, Fig. S2, Fig. S3)**. The aHIP network showed much stronger connectivity with widely distributed brain regions in AMYG, ACC, and ITG compared with the middle and posterior seeds. The mHIP network showed much stronger connectivity with CER compared with the anterior and posterior seeds. The pHIP network showed much stronger connectivity with widely distributed brain regions in THA, LOG, MOG, and CAL compared with the anterior and middle seeds. Notably, the subcortical region including THA, and cortical regions including LOG, MOG, and CAL showed a gradient increasing pattern on FC strength with seeds along the anterior through middle to posterior axis of the HIP, with the strongest connectivity of the posterior seed with those regions. Interestingly, these regions including the AMYG, ACC, and ITG showed a gradient decreasing pattern.

Supplementary tables

Table S1.

Neuropsychological data for all subjects

Items		HC n=129	aMCI n=85	*T* values (χ^2)	*p* values
**MDRS**					
Attention	raw score	36.34(0.85)	36.05(0.90)	2.417	0.017*
	Z score	0.13(0.97)	-0.20(1.02)	2.417	0.017*
Initiation/Preservation	raw score	36.43(1.81)	33.78(4.28)	6.241	0.000*
	Z score	0.32(0.55)	-0.49(1.30)	6.241	0.000*
Conceptual	raw score	37.29(1.60)	36.47(1.86)	3.460	0.001*
	Z score	0.19(0.91)	-0.28(1.06)	3.460	0.001*
Construct	raw score	5.44(0.87)	5.28(0.90)	1.294	0.197
	Z score	0.072(0.99)	-0.28(1.06)	1.294	0.197
Memory	raw score	22.42(1.41)	19.72(3.28)	8.270	0.000*
	Z score	0.40(0.53)	-0.61(1.22)	8.270	0.000*
Total	raw score	137.92(3.57)	131.29(6.85)	9.252	0.000*
	Z score	0.43(0.59)	-0.66(1.13)	9.252	0.000*
**Episodic memory**					
AVLT-20-min-DR	raw score	7.50(1.96)	2.34(1.63)	20.146	0.000*
	Z score	0.66(0.63)	-1.00(0.52)	20.146	0.000*
LMT-20-min-DR	raw score	8.33(2.43)	4.62(3.37)	9.322	0.000*
	Z score	0.44(0.72)	-0.60(0.92)	9.322	0.000*
ROCFT-20-min-DR	raw score	18.75(5.33)	11.61(6.47)	8.796	0.000*
	Z score	0.42(0.79)	-0.64(0.96)	8.796	0.000*
**Visuospatial function**					
ROCFT	raw score	34.31(1.65)	33.31(3.00)	3.148	0.002*
	Z score	0.17(0.71)	-0.26(1.1)	3.148	0.002*
CDT	raw score	8.89(1.21)	7.86(1.79)	5.036	0.000*
	Z score	0.26(0.78)	-0.40(1.12)	5.036	0.000*
**Information processing speed**					
DSST	raw score	39.28(10.35)	29.82(10.11)	6.602	0.000*
	Z score	0.33(0.92)	-0.50(1.17)	6.602	0.000*
TMT-A	raw score	66.66(18.71)	84.02(35.55)	-4.658	0.000*
	Z score	-0.25(0.67)	0.37(1.27)	-4.658	0.000*
Stoop-A	raw score	26.32(5.13)	32.48(8.66)	-6.535	0.000*
	Z score	-0.33(0.69)	0.50(1.17)	-6.535	0.000*
Stoop-B	raw score	39.95(10.37)	47.41(13.35)	-4.609	0.000*
	Z score	-0.24(0.85)	0.43(1.11)	-4.609	0.000*

**Executive function**					
VFT	raw score	26.07(5.71)	19.78(6.77)	7.323	0.000*
	Z score	0.36(0.83)	-0.55(0.99)	7.323	0.000*
DST-Backward	raw score	4.91(1.45)	4.33(1.28)	2.989	0.001*
	Z score	0.16(1.03)	-0.24(0.91)	2.989	0.001*
TMT-B	raw score	178.39(65.31)	255.45(123.36)	-5.947	0.000*
	Z score	-0.31(0.65)	0.46(1.23)	-5.947	0.000*
Stoop-C	raw score	80.17(21.67)	99.27(29.85)	-5.419	0.000*
	Z score	-0.28(0.81)	0.43(1.11)	-5.419	0.000*
Simliarity	raw score	19.49(3.20)	16.85(3.81)	5.507	0.000*
	Z score	0.29(0.86)	-0.43(1.04)	5.507	0.000*

Data are presented as the mean (standard deviation, SD). Abbreviations: MDRS, Mattis Dementia Rating Scale; AVLT-20-min-DR, Auditory Verbal Learning Test – 20-minute delayed recall; LMT-20-min-DR, Logical Memory Test – 20-minute delayed recall; ROCFT-20-min-DR, Rey-Osterrieth Complex Figure Test – 20-minute delayed delayed recall; CDT, Clock Drawing Test; ROCFT, Rey-Osterrieth Complex Figure Test; DSST, Digital Symbol Substitution Test; TMT-A, Trail Making Test-A; Stroop, Stroop Color and Word Test; VFT, Verbal Fluency Test; DST, Digit Span Test; TMT-B, Trail Making Test-B; Similarity, Semantic Similarity Test.

Table S2.

The mean (standard deviation, SD) rms of movement or rotation and SD of the signal before (pre) and after (post) removal of contaminated frames that contained large amounts of movement

	rms pre (mm)	rms pre (°)	SD pre	Percentage of frames removed	rms post (mm)	rms post (°)	SD post
aMCI	0.12(0.05)	0.06(0.04)	0.09(0.08)	4.2 (3.1)	0.09(0.05)	0.04(0.03)	0.14
HC	0.11(0.04)	0.07(0.05)	0.15(0.11)	6.8(4.2)	0.10(0.04))	0.04(0.02)	0.65

The mean rms decreased after this procedure with differences not significantly affected by group membership.

Supplementary figures

Figure S1.

Resting-state functional connectivity patterns within group maps of the distinct MTL subregions.

A) showing the subregions defined in MTL. B, C) representing the within-group statistical maps of HCs and aMCI patients, respectively, with statistical threshold set at $p_{corrected} < 0.01$, corrected by FEW and cluster extent k > 100 voxels (800 mm^3) . Color bar is presented with z score, respectively. Notes: HC, healthy control; L, the left MTL subregional networks; R, the right MTL subregional networks; FC, functional connectivity.

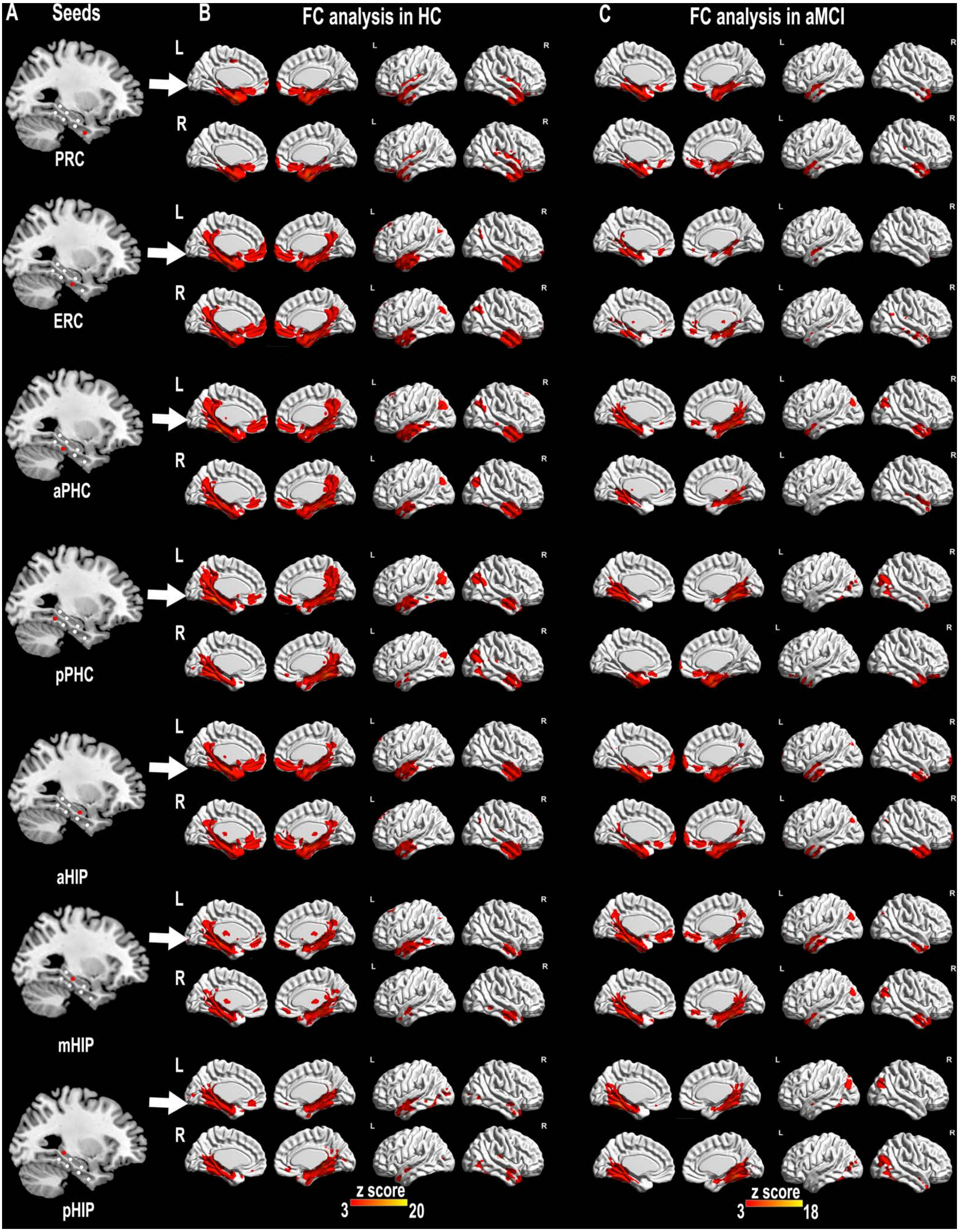
A
Seeds
B
FC analysis in HC
C
FC analysis in aMCI
PRC
ERC
aPHC
pPHC
aHIP
mHIP
pHIP
L
R
z score
3
20
z score
3
18

Figure S2.

Heterogeneous functional connectivity associated with four distinct parahippocampal and three distinct hippocampal seeds in HC subjects.

A) Schematic polar plot depicts connectivity patterns of four parahippocampal seeds with target ROIs distributed across the whole brain in HC subjects. The concentric circles depict parameter estimates representing the connectivity strength. B) Schematic polar plot depicts connectivity patterns of three hippocampal seeds with target ROIs distributed across the whole brain in HC subjects. Note that the data of functional connectivity are extracted from the only brain regions corresponding to Fig. S2. Abbreviation: AMYG, amygdala; THA, thalamus; ACC, anterior cingulate gyrus; PUT, putamen; PCUN, precuneus; PCC, posterior cingulate cortex; LOG, inferior occipital gyrus; MOG, middle occipital gyrus; CUN, cuneus; CAL, calcarine gyrus; SFG, superior frontal gyrus; REG, rectus gyrus; ITG, inferior temporal gyrus; STG, superior temporal gyrus; MTG, middle temporal gyrus; CER, cerebelum; HIP, hippocampus.

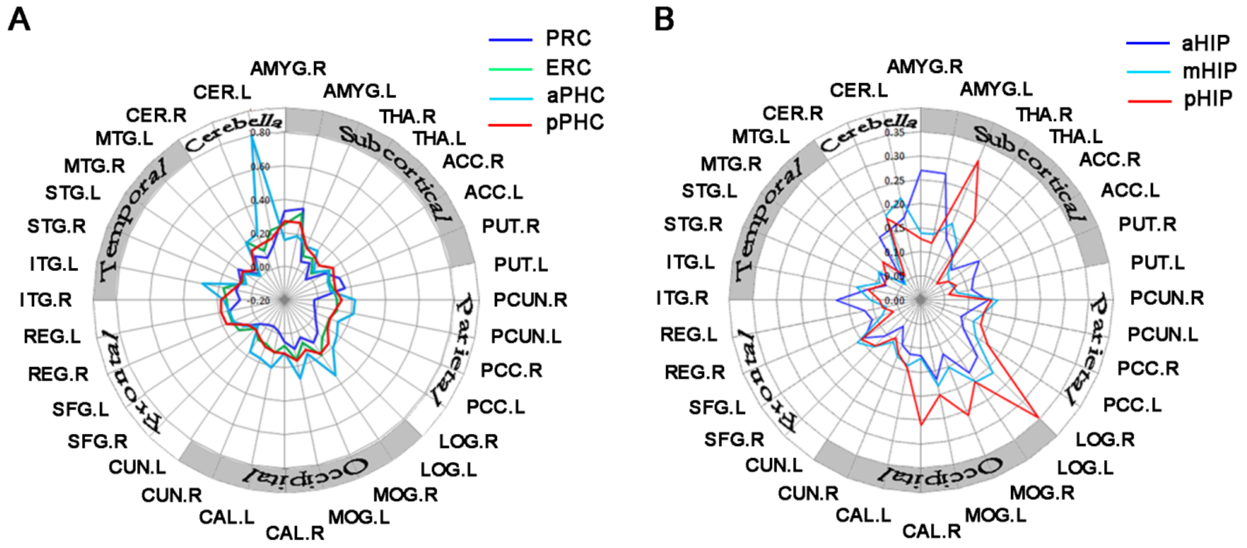

Figure S3.

Correlation matrix within seven seed regions of interest in the left and right hemispheres.

A, B) representing the correlation matrix estimated by calculating the Pearson's correlation coefficients between each pair of the within group statistical maps of MTL subregions in HC (A) and aMCI (B) subjects. Notes: aHIP, anterior hippocampus; mHIP, middle hippocampus; pHIP, posterior hippocampus; PRC, perirhinal cortex; ERC, entorhinal cortex; aPHC, anterior parahippocampal cortex; pPHC, posterior parahippocampal cortex.

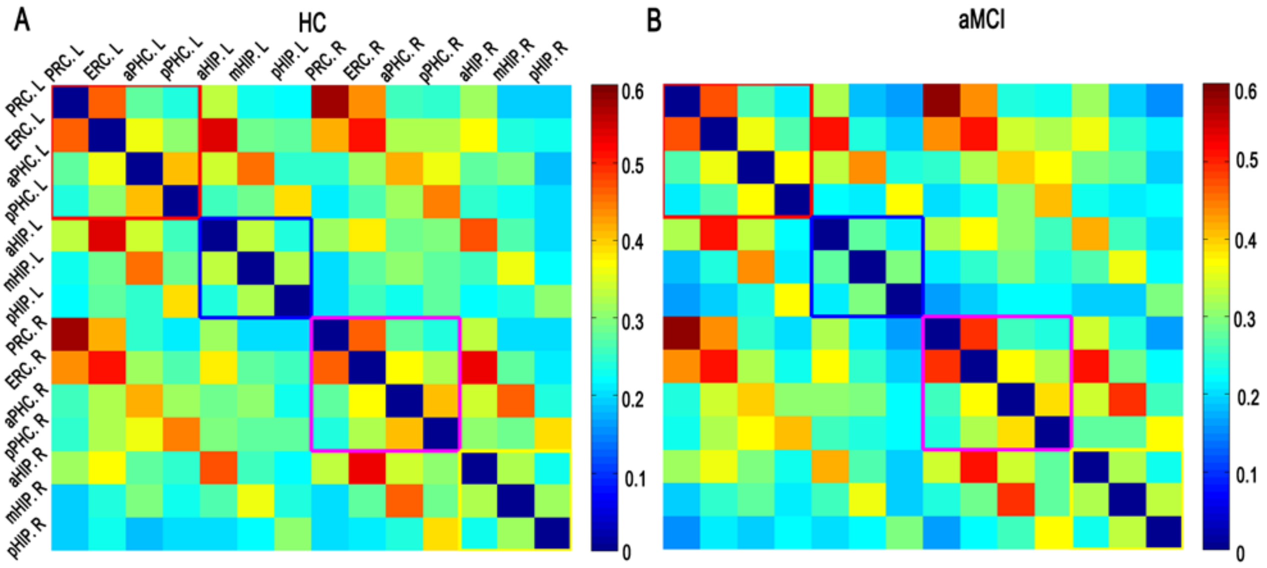

Figure S4

Results from the analysis of voxel-based morphometry in Statistical Parametric Maps (SPM) for GM volume in the whole brain at a threshold of $p < 0.05$ using FDR correction and cluster extent k > 320 mm^3.

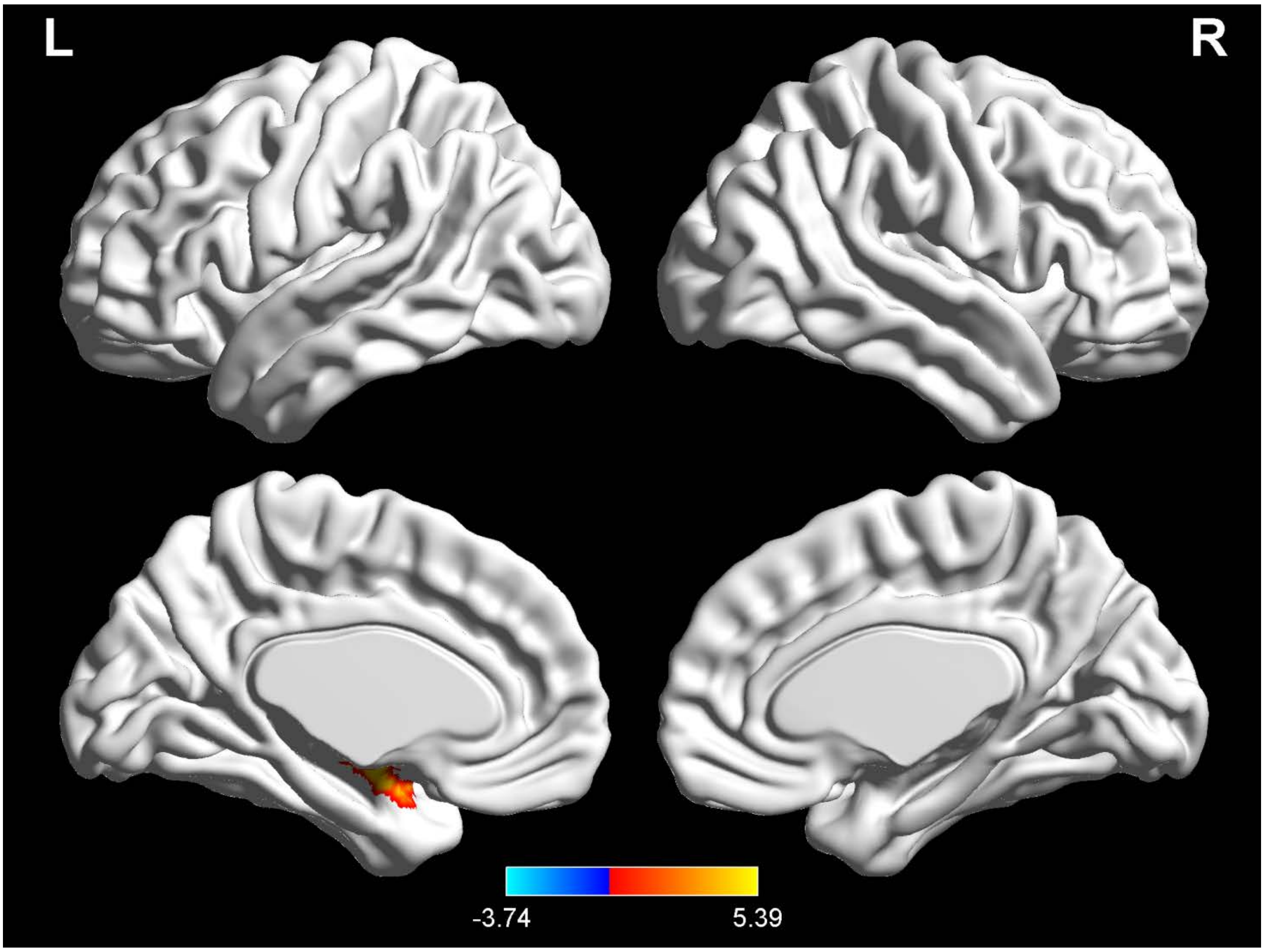

Figure S5, related to Figure 3

Influence of methodological changes regarding the identification of distinct altered FC networks of MTL subregions.

For the sake of comparison, results presented in the main article are also presented in this figure, including A) presenting the seeds of MTL subregions for FC analysis (identical to Figure 3A). In order to the aesthetics, only the seeds in the left hemisphere were displayed. B) presenting the comparison of HC and aMCI patients using gray matter atrophy corrected (GMAc) and head motion effect corrected (HMEc) (identical to Figure 3B). C, D) presenting the comparison of HC and aMCI patients without the use of GMAc and HMEc, respectively.

The two sets of complementary analyses include FC comparison between HC and aMCI patients without the use of GMAc (C) and HMEc (D).

Using the same preprocessing step and statistical threshold ($p_{uncorrected} < 0.005$ and cluster extent k > 80 (640 mm^3), corresponding to thresholds of AlphaSim-corrected $p < 0.05$), results appear to be very similar, though generally more significant than when using GMAc FC data and with clusters being generally larger. In addition, a new set of regions located in bilateral lingual gyrus, and bilateral precuneus. However, despite this discrepancy, it is worth highlighting that the same brain regions of distinct altered FC networks of MTL subregions were recovered in this analysis.

In conclusion, while the method slightly impacted the identification of brain regions of distinct altered FC networks of MTL subregions, the functional changes of the brain regions and neuronal circuit in MTL subregions highlighted in the main manuscript were found in both complementary analyses and can therefore be considered as a strong and stable finding.

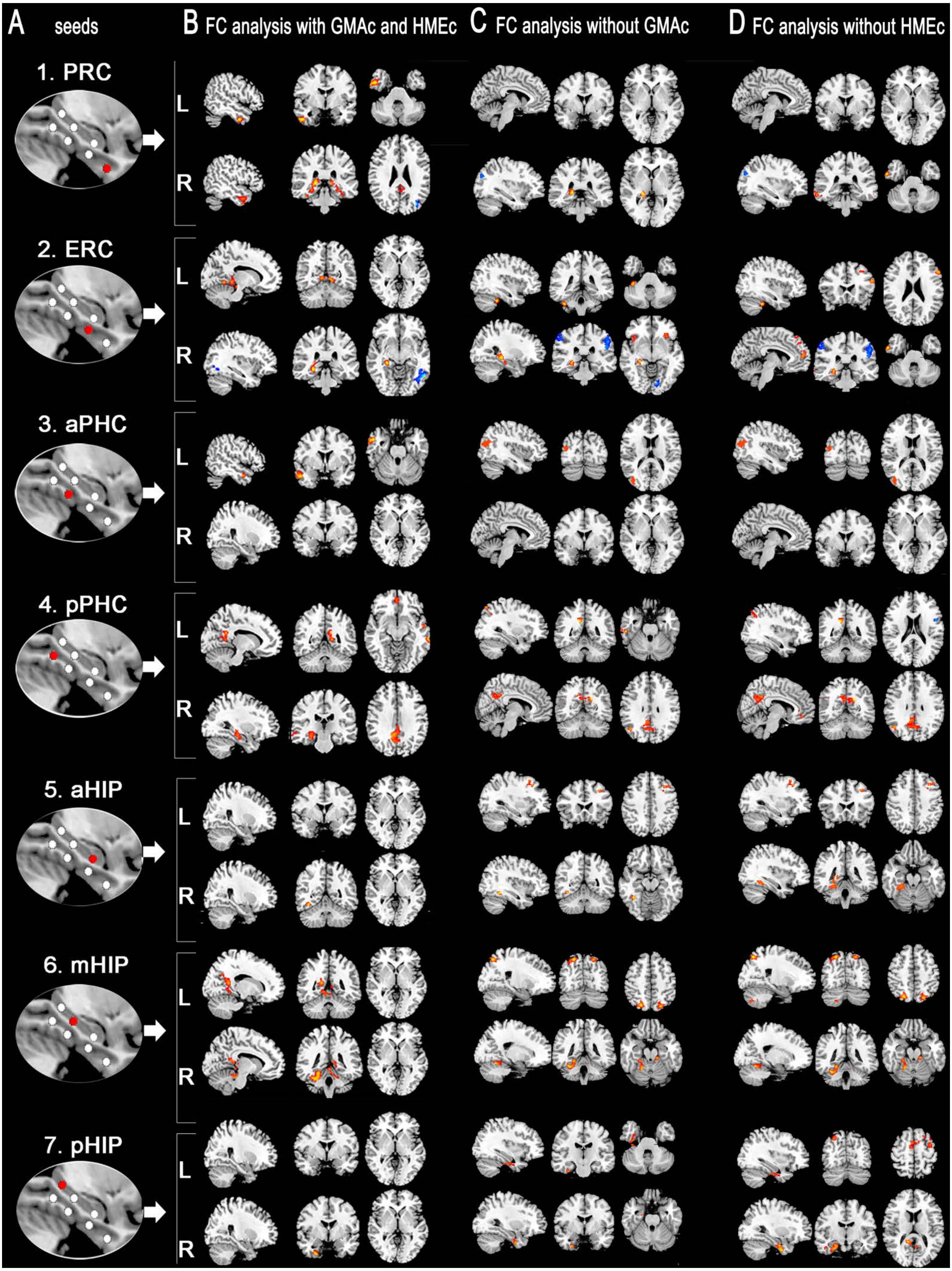
A seeds
B FC analysis with GMAc and HMEc
C FC analysis without GMAc
D FC analysis without HMEc
1. PRC
2. ERC
3. aPHC
4. pPHC
5. aHIP
6. mHIP
7. pHIP
L
R

Reference

1 Petersen, R. C. *et al.* Mild cognitive impairment: clinical characterization and outcome. *Arch Neurol* **56**, 303-308 (1999).

2 Winblad, B. *et al.* Mild cognitive impairment--beyond controversies, towards a consensus: report of the International Working Group on Mild Cognitive Impairment. *J Intern Med* **256**, 240-246 (2004).

3 Shu, H. *et al.* Opposite Neural Trajectories of Apolipoprotein E 4 and 2 Alleles with Aging Associated with Different Risks of Alzheimer's Disease. *Cereb Cortex* (2014).Doi: 10.1093/cercor/bhu237.

4 Xie, C. *et al.* Abnormal insula functional network is associated with episodic memory decline in amnestic mild cognitive impairment. *Neuroimage* **63**, 320-327 (2012).
